# The Influence of Gain and Loss on Arithmetic Performance

**DOI:** 10.3389/fpsyg.2017.02150

**Published:** 2017-12-12

**Authors:** Ram Naaman, Liat Goldfarb

**Affiliations:** Edmond J. Safra Brain Research Center for the Study of Learning Disabilities, Department of Learning Disabilities, University of Haifa, Haifa, Israel

**Keywords:** arithmetic performance, gain, loss, reward, arithmetic problems, numerical cognition, addition problems, reward modulation

## Abstract

Gain and loss modulation of different aspects of executive functions (EF) has been studied under changing conditions. However, the nature of this effect varies in different EF tasks, as both gain and loss were found to improve performance in specific EF tasks while hindering performance in others. The current study examines the influence of gain and loss stimuli on arithmetic performance. Since arithmetic processes have been found to rely heavily on EF, the current study addresses the question of “whether” and “in what direction” those stimuli might affect arithmetic performance. In three experiments, participants preformed an arithmetic equation judgment task, while gain and loss conditions were added in each trial in the form of a line drawn face representing either monetary gain, loss, or neither. In Experiment 1, the arithmetic task included carry and non-carry equations representing different arithmetic complexity levels. In Experiment 2, two and three addend equations were used, and in Experiment 3, the proportions of correct and incorrect equations differed. Results of all experiments demonstrated faster RT in the arithmetic task after gain stimuli when compared to the loss stimuli. Our results further extend our understanding regarding the nature of the relationship between gain and loss situations and arithmetic performance and further emphasize the conditions under which arithmetic performance can be improved or hindered.

## Introduction

Learning to perform arithmetic is one of the most fundamental abilities acquired during our formal education (e.g., Durand et al., 2005). Arithmetic abilities are thought to be crucial not only during our school years but for almost every aspect of our daily life (e.g., shopping, calculating small change, and paying bills). Arithmetic include basic mathematical operations such as addition, subtraction, multiplication, and division. Different complexities of arithmetic require different processes to various extents. That is, while simple forms of arithmetic calculation (e.g., 5×4 or 3+2) usually rely on retrieving the correct solution from long term memory, other, more complex forms of arithmetic calculation (e.g., 46+29) usually require complex processes involving various cognitive mechanisms (e.g., Passolunghi and Siegel, 2001; Espy et al., 2004; St Clair-Thompson and Gathercole, 2006). Among these cognitive mechanisms, executive functions (EF) were found to be closely related to different arithmetic operations, having a major role in their developmental trajectory and normal functioning (Espy et al., 2004).

### The Role of Executive Functions in Arithmetic

Executive functions are considered an umbrella term for various cognitive abilities responsible for regulating goal directed behavior, especially in novel or demanding situations. These various abilities include inhibition, shifting, and working memory (WM), among others (e.g., Huizinga et al., 2006; Banich, 2009). As a result, EF have a crucial impact on our ability to work, study, function independently, and maintain appropriate social relations (Chan et al., 2008; Kofler et al., 2011). Accumulated findings from different studies suggest that the prefrontal cortex, and especially the dorsolateral prefrontal cortex (DLPFC) and the anterior cingulate cortex (ACC), are involved in many EF operations (e.g., Huizinga et al., 2006; Jurado and Rosselli, 2007; Banich, 2009).

Numerous studies performed on a variety of age groups found numerical and arithmetic abilities to be strongly linked to EF. These include shifting, inhibition, WM, and other cognitive skills such as immediate recall of numerical information (e.g., Bull and Scerif, 2001; Passolunghi and Siegel, 2001; De Stefano and LeFevre, 2004; van der Sluis et al., 2004; Merkley et al., 2016). Studies investigating the developmental trajectory of EF have shown a significant development in WM, shifting, and inhibition during preschool and elementary school years (Siegel and Ryan, 1989; Espy, 1997; Hughes, 1998). During that same time period, major changes are also found in counting skills and problem solving, among other arithmetic abilities (e.g., Resnick, 1989; Ostad, 1998). The shared developmental trajectory supports the numerous findings regarding a close connection between the two. In support of this notion, Espy et al. (2004) examined a group of 96 preschoolers for various EF and arithmetic skills, and found that WM as well as inhibitory control significantly predicted arithmetic skills in preschool children.

One EF repeatedly linked to arithmetic is the WM, a limited capacity system responsible for temporal storing and processing of information. It is considered to be strongly affected by specific factors such as the amount of items stored or the number of tasks executed simultaneously (e.g., Baddeley, 2000; Fürst and Hitch, 2000; Imbo et al., 2007). Regarding arithmetic abilities, the WM plays a key role in single and multiple digit arithmetic. Arithmetic operations (i.e., addition, subtraction, multiplication, and division) usually involve the temporal storage of intermediary results. This information is stored temporarily in the WM during calculation and then retrieved further on in the process in order to complete the calculation (De Stefano and LeFevre, 2004; Imbo et al., 2007). Temporal storage and processing of information makes the WM, as well as inhibition of no longer needed information, crucial for arithmetic calculations. Furthermore, the involvement of WM in arithmetic grows as a function of the calculation’s complexity, resulting from the need to process larger amounts of data and perform multiple mental steps during calculations (De Stefano and LeFevre, 2004; Raghubar et al., 2010). An example of this differentiation in complexity may be seen in carry vs. non-carry equations. In carry equations, the sum of either or all positions (units, tens, hundreds, etc.) of both respective addends is equal to or higher than 10 (e.g., 28+45 or 733+692). In these situations, the solution process obligates the holding of an interim result before adding it to the final solution. For example, when calculating 28+45 the sum of the units 8+5 equals 13. The carry operation is then performed by adding 1 to the sum of the tens of the two addends (i.e., 2+4+1 = 7), leaving the units with the sum of 3. Finally, the interim results of both the tens (7) and the units (3) are then added to form the final solution of 73. On the other hand, in non-carry equations, neither sum of any of the positions (units, tens, hundreds, etc.) of both respective addends equals 10 or higher (e.g., 24+42), resulting in a simpler calculation. The process of holding an interim result when performing carry operations relies heavily on different EF, among them WM and inhibition. Therefore, these operations seem to form a cognitive load, depending on the number of carry operations needed and their sum. That is, the more carry operations needed and the larger their sum, the more reliance they demand on EF mechanisms (Fürst and Hitch, 2000; De Stefano and LeFevre, 2004; Imbo et al., 2007).

### Modulating EF Performance Using Gain and Loss Stimuli

As noted above, EF are crucial for our daily life and proper functioning in various activities, including arithmetic. A large body of literature has been devoted to examining different cognitive and emotional factors that modulate and enhance EF performance. One method shown to successfully modulate EF performance comes from the field of emotion and its influence on EF. It has been suggested that activation of various neuronal networks in the frontal lobe is mediated by different emotional states through mechanisms of the dopaminergic system (e.g., Nieoullon and Coquerel, 2003). Among these emotional states, the processing of experienced gains and losses (or rewards and punishments) seem to play an important role in modulating cognition, especially in situations involving EF operations such as cognitive control (e.g., van Steenbergen et al., 2009; Krebs et al., 2010; Braem et al., 2012). For example, an fMRI study conducted by Gilbert and Fiez (2004) examined the affective modulation of gain stimuli (monetary reward) on WM and levels of activation in the DLPFC. In their study, participants were scanned while performing a serial delayed recall task. Each trial began with either a reward or non-reward cue, signaling the potential gain in the current trial. Following the monetary cue, a five-word list (level of difficulty was manipulated by controlling for the number of syllables) was presented, followed by a delayed phase and a recall phase. Behavioral results showed higher accuracy levels for rewarded trials as opposed to unrewarded trials. More importantly, the fMRI findings showed higher DLPFC activation for rewarded trials than for unrewarded trials. Interestingly, while higher task complexity led to increased DLPFC activity on one hand and to a decrease in performance on the other, reward signals led to both increased DLPFC activity and task performance.

Another behavioral example of the impact of gain and loss stimuli on EF can be seen in the context of inhibition and cognitive control. However, in this case gain stimuli had a negative impact on EF. van Steenbergen et al. (2009) examined the effect of reward (gain stimuli) on inhibition. Their study used an adjusted flanker task with line drawn happy, sad, or neutral faces. Each flanker stimuli was followed by the presentation of a face independent of the participant’s performance (e.g., a smiley face could appear after either correct or incorrect responses). Participants were informed that the faces would be presented randomly and independent of their performance and that they would earn extra money for each happy face presented and lose money for a sad one. Their results show that when compared to loss stimuli (i.e., loss of money), reward stimuli (i.e., gaining bonus money) led to a decrease in cognitive control and inhibition, reflected by a reduction in the conflict adaptation effect in the following trial. van Steenbergen et al. (2009) suggested that conflicting situations are experienced as aversive events leading to enhanced recruitment of cognitive control processes. Hence, they argued that unexpected monetary gain, representing motivation for a positive emotional state, might collide with the effect of aversive conflict and thus diminish the enhancement of cognitive control expected after conflictive events. They concluded that the observation of two emotional states that cancel each other points toward a shared basis and offered the dopaminergic system as the mechanism underlying it. These findings support the notion that a positive emotional state might hinder performance in cases that require inhibition of unrelated information.

Gain and loss situations were also examined in the context of complex problem solving (CPS). In a study by Barth and Funke (2010), participants performance in a CPS task was examined either in a positive environment, enabling higher profits, or a negative environment with harder profit conditions. According to their results, participants operating in negative environments displayed better overall performance in the CPS task and better analytic processing. These results are in partial contrast to an earlier study by Spering et al. (2005), who used negative and positive feedbacks in a similar CPS task. In their study, different emotional feedback did not seem to differentially influence participants’ overall performance in the task aside form a tendency toward a better information gathering and more detailed information search after negative feedback.

To sum up, a large body of literature suggests that gain and loss stimuli may modulate EF. While some studies suggest that gain stimuli have a positive influence on different aspects of EF, others suggest that it has a negative influence on other aspects of EF (see also Braem et al., 2013; Chiew and Braver, 2014). Another body of literature suggests that EF plays a crucial role in arithmetic. Nevertheless, the connection between arithmetic and gain and loss situations has never been studied. Therefore, the current work will examine the effect of gain and loss stimuli on arithmetic performance.

The current study includes three experiments in which we examined whether gain and loss stimuli will differentially modulate arithmetic performance. In all experiments, participants preformed an arithmetic equation judgment task under different gain/loss conditions. Each arithmetic equation was followed by a line drawn face representing either gain (happy face), loss (sad face), or neutral conditions (neutral face). In Experiment 1, we used an arithmetic equation judgment task with either carry or non-carry equations representing different complexity levels. In Experiment 2, we used different levels of complexity using two or three addend equations. Finally, in Experiment 3 we manipulated the proportion of correct–incorrect equations.

## Experiment 1

Experiment 1 examined the effect gain and loss stimuli have on arithmetic performance. In this experiment, carry and non-carry equations were followed by a line drawn face representing either gain, loss, or neutral conditions. As noted above, more carry operations lead to heavier reliance on EF mechanisms (Fürst and Hitch, 2000; De Stefano and LeFevre, 2004; Imbo et al., 2007). Hence, the current design allowed us to examine whether gain and loss stimuli will differentially modulate arithmetic performance and whether this influence is affected by the different levels of task complexity.

### Materials and Methods

#### Participants

Fourteen university students (mean age = 28.07; *SD* = 4.06; 9 females) participated in this experiment in return for course credits or payment ($7). Participants were informed about the possibility of earning up to an extra $2 if they get lucky and regardless of their performance on the task. All participants were native Hebrew speakers with no declared learning disabilities or ADHD. The inclusion criterion for all experiments was normal performance (in RT) on the arithmetic task (within ±2 standard scores and no more than 15% error rate).

#### Stimuli

**FIGURE 1:**
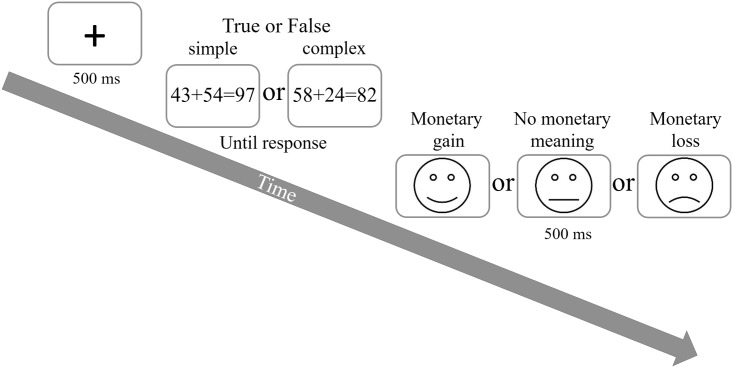
Trial procedure. Participants had to calculate and judge whether the equation presented is true or false by pressing one of two response buttons. Each trial was followed by one of three line drawn faces.

The arithmetic task consisted of double digit, two addend addition equations. These were written in Arial font, size 26, and were presented in the center of the computer screen. All equations were constructed according to Klein et al. (2010), with the following rules: the sum of each equation ranged from 61 to 99; multiples of 10 and ties (e.g., 44, 55, 66) were not included as addends or sums; the units and tens of each two addends were never identical. The line drawn faces following each equation were 4.5 cm in diameter, presented in the center of the screen. The different gaining conditions were expressed by the angle of the curve on the face’s mouth (smiling for gain, sad for loss, and a straight line for neutral, see **Figure 1**). All stimuli (equations and faces) were printed in black font and were presented on a white background.

#### Procedure

The computerized experimental task was designed by using E-Prime software (E-Prime 2.0, Psychology Software Tools, Pittsburgh, PA, United States). The task was administered using an *hp compaq* computer with an Intel i7 core processor. Stimuli were presented on an LCD Samsung SyncMaster SA350 screen (screen size: 27 inches, screen resolution: 1920 × 1080). A keyboard on which participants conveyed their answer was placed on a table next to the screen. All participants sat approximately 60 cm from the computer screen and were tested individually. Each experimental session began with instructions presented on the computer screen.

The current procedure was a modification of van Steenbergen et al.’s (2009) gain and loss experiment. In their experiment, they used the gain and loss manipulation on the Flanker task. Here we maintained a similar procedure regarding the gain and loss manipulation but changed the flanker task to an arithmetic task. Participants were informed that, following each arithmetic equation, a line drawn face would appear indicating either a gain of $0.15 (i.e., the appearance of a happy face), a loss of $0.15 (i.e., the appearance of a sad face) or no monetary meaning (i.e., the appearance of a neutral face). Importantly, they were also informed that the faces would be presented randomly and irrelevantly of their performance (e.g., a happy face could appear following either a correct response or an incorrect one, a slow response or a fast one).

Each trial began with a fixation cross for 500 ms. Following the fixation cross, an arithmetic equation was presented, with either a correct or an incorrect sum. Each equation was presented until response. Immediately after the participant’s response, a line drawn face was presented for 500 ms, after which the next trial started. Participants were required to solve the equations as quickly and accurately as possible, deciding whether their sum is true or false by pressing one of two response keys (i.e., the “M” key on the keyboard for correct equations and the “C” key for incorrect ones). Response time, as well as accuracy, were measured by the computer.

The arithmetic task included 266 equations in three blocks. The first block was a practice block including six equations. The second block served as the experimental block and included 240 equations. In this block, 50% (120) of the equations were carry and the rest were non-carry equations. In each level of difficulty (i.e., carry and non-carry), 80% (96) of the equations were correct and 20% (24) were incorrect. The proportions of correct and incorrect equations in arithmetic verifications tasks varies across different studies and experimental settings, ranging from 20 to 50% incorrect equations (Rivera et al., 2005; Klein et al., 2010; Jasinski and Coch, 2012; Cipora et al., 2013; Abramovich and Goldfarb, 2015). The use of 20% incorrect equations was chosen in this experiment in order to prevent strategy use and cognitive loading. When the proportion of incorrect trials is high, participants can rely on strategy use such as guessing or calculating only the tens or units. In addition incorrect equations were found to involve multiple cognitive processes that attribute to cognitive load (Menon et al., 2002; Menon, 2010). The third block included 20 equations and served as a filler block with overrepresentation of happy faces allowing all participants to earn the bonus money. This block was not included in the analysis. All face types, as well as equation correctness (correct vs. incorrect) and level of difficulty (carry vs. non-carry), were randomized and counter balanced across the experimental block.

### Results and Discussion

Incorrect trials, as well as trials with outlier RTs (±2 standard scores and under 300 ms), were excluded (overall, 7.86% of all trials). For the remaining trials, Mean RT under the different conditions was calculated for each subject. On this data, a repeated measures three-way ANOVA was performed, with gaining condition (RT for equation task after gain, loss, or neutral stimuli), equation difficulty (carry or non-carry), and equation correctness (equations with correct or incorrect sum displayed) as within subject independent variables.

**FIGURE 2:**
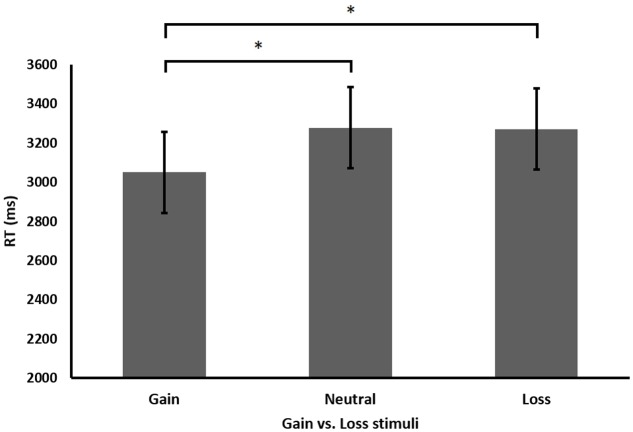
Performance (RT in milliseconds) in the different gaining conditions in Experiment 1. ^∗^*p* = 0.05.

As predicted, participants performed significantly faster when solving non-carry compared to carry equations *F*(1,13) = 30.193, *MSE* = 1.531, *p* < 0.001, and when solving correct compared to incorrect equations *F*(1,13) = 15.412, *MSE* = 7.635, *p* < 0.01. A marginally significant main effect for gaining condition (i.e., gain, neutral, and loss) was found (see **Figure 2**) *F*(2,26) = 2.964, *MSE* = 3.18, *p* = 0.069. Significant interactions were found between equation difficulty and equation correctness *F*(1,13) = 4.788, *MSE* = 9.818, *p* < 0.05, between equation correctness and gaining condition *F*(2,26) = 4.319, *MSE* = 1.855, *p* < 0.01, and between equation difficulty and gaining condition, *F*(2,26) = 4.682, *MSE* = 1.414, *p* < 0.025. The three-way interaction between equation correctness, equation difficulty, and gaining condition was also found significant *F*(2,26) = 3.978, *MSE* = 1.323, *p* < 0.05. Importantly, further analysis of the main effect of the gaining condition revealed that beyond all conditions gain stimuli facilitated the following arithmetic task compared to loss and neutral stimuli, *F*(1,13) = 11.092, *MSE* = 169903, *p* < 0.01. Furthermore, RT in the arithmetic task after gain stimuli was faster when compared to loss stimuli, *t*(13) = 2.15, *p* = 0.05. Moreover, performance after gain stimuli was also faster when compared to neutral stimuli *t*(13) = 2.754, *p* < 0.025. No significant differences were found between loss and neutral stimuli, *t*(13) = 0.0572, *p* = 0.955. Further analysis was conducted of the two-way interactions between the gaining condition and equation difficulty or correctness. The findings suggest that the advantage of gain stimuli over loss stimuli was significantly larger in the carry condition (Mean difference = 410) than in the non-carry condition (Mean difference = 31), *F*(1,13) = 11.079, *MSE* = 90520, *p* < 0.01, and marginally larger in the incorrect (Mean difference = 350) compared to correct (Mean difference = 92) equations, *F*(1,13) = 4.296, *MSE* = 108823, *p* = 0.058. In addition, analyzing the accuracy rate in each condition revealed no significant results, all *p*’s > 0.05.

To sum up, we hypothesized that gain and loss stimuli will differentially affect arithmetic performance. Interestingly, results show that gain stimuli led to faster RT compared to loss stimuli, and when compared to neutral stimuli. This pattern was emphasized when carry operations were needed for the solution.

## Experiment 2

The purpose of this experiment was to further examine whether gain stimuli, when compared to loss stimuli, lead to faster RT in the arithmetic task on different types of arithmetic addition problems. In this experiment, rather than using carry and non-carry two addend equations, participants were asked to solve arithmetic equations of different complexity levels, manipulated by the number of addends in each equation (two and three addends). In multiple step arithmetic, the holding and processing of intermediate results is required (e.g., Raghubar et al., 2010). For example, when solving the equation 2+4+3, adding the interim result of 6 (2+4) to the third addend results in the final sum of 9. Moreover, the more steps required for the solution, the higher the risk of calculation errors as a result of the cognitive load imposed (Ayres, 2001). Furthermore, the information of the interim results must not only be maintained but also inhibited later on in the process in order to efficiently and correctly perform the calculation (Abramovich and Goldfarb, 2015).

### Materials and Methods

The method in Experiment 2 was similar to that of Experiment 1, aside from the following changes: 14 university students (mean age = 25.64; *SD* = 2.79; 12 females) participated in this experiment. In Experiment 2, each equation could be the sum of either two or three addends. In order to control for other aspects of equation complexity, all equations were of the non-carry type and sums ranged from 61 to 99, resulting in different equations for similar sums (e.g., 31+47 = 78 or 15+42+21 = 78).

### Results and Discussion

Incorrect trials, as well as trials with outlier RTs (±2 standard scores and under 300 ms), were excluded (overall 9.48%). For the remaining trials, mean RT in the different conditions was calculated for each participant. On this data, a repeated measures three-way ANOVA was performed, with gaining condition (gain, loss, or neutral stimuli), equation difficulty (two or three addends), and equation correctness (equation with correct or incorrect sum displayed) as within subject independent variables.

**FIGURE 3:**
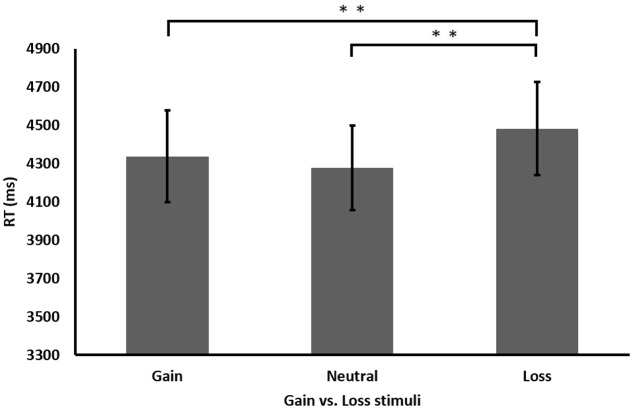
Performance (RT in milliseconds) in the different gaining conditions in Experiment 2. ^∗∗^*p* < 0.01.

The results revealed that participants performed significantly faster when solving two addend compared to three addend equations, *F*(1,13) = 114.42, *MSE* = 2.158, *p* < 0.001, as well as when solving correct equations compared to incorrect ones, *F*(1,13) = 9.616, *MSE* = 4.09, *p* < 0.01. Of special interest is the significant main effect found for gaining condition, suggesting differential performance as a result of the gain, neutral, or loss stimuli, *F*(2,26) = 6.076, *MSE* = 1.028, *p* < 0.01 (see **Figure 3**). No other significant interactions were found, all *p*s > 0.05.

Further analysis of the gaining condition’s main effect revealed an advantage for gain stimuli, with participants performing significantly faster after gain stimuli than after loss stimuli, *t*(13) = 2.392, *p* < 0.01. However, examining the relationship with the neutral stimuli revealed a different pattern than those seen in the previous experiment. That is, participants’ performance was faster also after neutral stimuli compared to loss stimuli, *t*(13) = 3.673, *p* < 0.01. No differences were found between gain and neutral stimuli, *t*(13) = 0.926, *p* = 0.371. As in Experiment 1, analyzing the accuracy rate in each condition revealed no significant results, all *p*’s > 0.05.

To sum up, the findings from Experiment 2 replicated the findings from Experiment 1 regarding faster RT following gain stimuli compared to loss stimuli. In contrast to Experiment 1, this time no interaction was found between gaining condition and equation difficulty.

## Experiment 3

The purpose of this experiment was to further replicate the findings that gain stimuli lead to faster RT compared to loss stimuli, in another type of experimental setting. In Experiments 1 and 2 the proportion between the display of correct and incorrect equations was 80/20, while in the current experiment the proportions were changed to 50/50.

### Materials and Methods

The method in Experiment 3 was similar to that of Experiment 2, aside from the following changes: 14 university students (mean age = 25.71; *SD* = 4.68; 10 females) participated in the experiment. As mentioned above, here we changed the proportion of correct and incorrect equations in the arithmetic task to 50/50 (120 trials for each condition).

### Results and Discussion

Incorrect trials, as well as trials with outlier RTs (±2 standard scores and under 300 ms), were excluded (overall, 11.94%). For the remaining trials, mean RT under the different conditions was calculated for each subject. On this data, a repeated measures three-way ANOVA was performed, with gaining condition (gain, loss, or neutral stimuli), equation difficulty (two or three addends), and equation correctness (equation with correct or incorrect sum displayed) as within subject independent variables.

**FIGURE 4:**
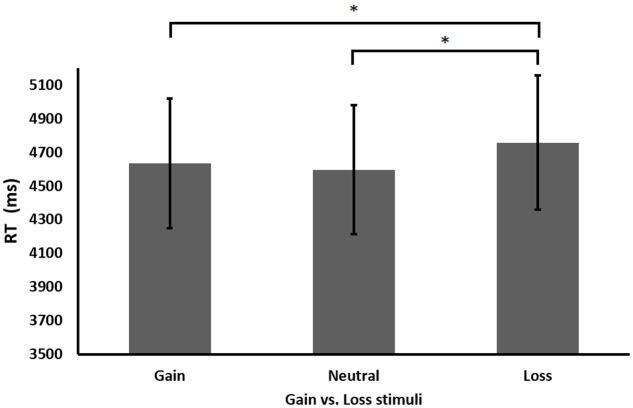
Performance (RT in milliseconds) in the different gaining conditions in Experiment 3. ^∗^*p* < 0.05.

As expected, participants solved two addend equations significantly faster than three addend equations, *F*(1,13) = 104.435, *MSE* = 2.65, *p* < 0.001. More interestingly, as seen in Experiment 2, here too a significant main effect was found for gaining condition, *F*(2,26) = 3.740, *MSE* = 1.076, *p* < 0.05 (see **Figure 4**). Additionally, an interaction was found between equation difficulty and equation correctness, *F*(1,13) = 8.588, *MSE* = 2.32, *p* < 0.025. No other main effects or interactions were found, all *p*s > 0.05. Lastly, analyzing the accuracy rate in each condition revealed no significant results, all *p*’s > 0.05.

Further analysis of the significant gaining condition main effect revealed that participants’ performance was again significantly faster after gain stimuli than after loss stimuli, *t*(13) = 1.83, *p* < 0.05. Furthermore, as seen in Experiment 2, performance after neutral stimuli was significantly faster than after loss stimuli, *t*(13) = 2.54, *p* < 0.025, but did not differ from gain stimuli, *t*(13) = 0.713, *p* = 0.488.

To sum up, the findings from Experiment 3 replicated the pattern regarding faster RT after gain stimuli compared to loss stimuli.

### Joint Analysis: Experiments 1–3

In Experiments 1–3, we examined the effect of gain and loss on performance in an arithmetic equation judgment task. In this joint analysis, we combined all three experiments in order to see general patterns beyond the specific experiments.

For the purpose of analyzing the combined data of Experiment 1 with Experiments 2 and 3, we used a four way repeated measures ANOVA with gaining condition (gain, loss, or neutral stimuli), equation difficulty (“simple” = non-carry or 2 addend equations vs. “complex” = carry or 3 addend equations), equation correctness (correct vs. incorrect equations) as within subject independent variables and “experiment” (1, 2, or 3) as the between subject independent variable.

The joint analysis revealed a significant main effect for equation difficulty, *F*(2,39) = 241.766, *MSE* = 2.113, *p* < 0.01, equation correctness, *F*(1,39) = 14.682, *MSE* = 6.323, *p* < 0.001, and experiment, *F*(2,39) = 6.976, *MSE* = 1.438, *p* < 0.01. Importantly, the combined analysis yielded a significant main effect for gaining condition, *F*(2,78) = 6.837, *MSE* = 1.762, *p* < 0.01. In addition, a significant interaction was found between equation difficulty, correctness, and experiment, *F*(2,39) = 4.762, *MSE* = 2.667, *p* < 0.025, and between equation difficulty and experiment, *F*(2,39) = 13.961, *MSE* = 2.113, *p* < 0.01, suggesting that participants in the different experiments differed in their performance under the different complexity levels. No other main effects or interactions were found, all *p*s > 0.05.

**FIGURE 5:**
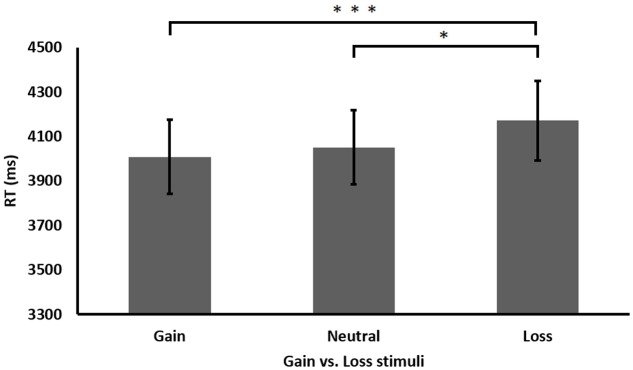
Performance (RT in milliseconds) in the different gaining conditions. Performance after loss stimuli was slower than after gain or neutral stimuli. ^∗^*p* < 0.05, ^∗∗∗^*p* < 0.001.

Further analysis of the significant main effect of gaining condition revealed that participants performed faster after gain stimuli than after loss stimuli, *t*(41) = 3.572, *p* < 0.001, across all experiments (see **Figure 5**). Moreover, participants also performed faster after neutral stimuli than after loss stimuli, *t*(41) = 2.332, *p* < 0.05. No significant differences were found between gain and neutral stimuli, *t*(41) = 0.997, *p* = 0.324. In addition, further analysis of the main effect of experiment revealed that performance in Experiment 1 (*M* = 3201, *SE* = 292) was significantly faster than in Experiment 2 (*M* = 4366, *SE* = 292), *t*(27) = 3.576, *p* < 0.01 or 3 (*M* = 4663, *SE* = 292), *t*(27) = 3.249, *p* < 0.01. No differences in performance were found between Experiments 2 and 3, *t*(27) = 0.655, *p* = 0.518. Moreover, further analysis of the interaction between equation difficulty and experiment found that under the complex condition (carry in Experiment 1 and three addends in Experiments 2 and 3), participants in Experiment 1 performed significantly faster (*M* = 3725, *SE* = 315) than participants in Experiment 2 (*M* = 5579, *SE* = 323), *t*(26) = 4.105, *p* < 0.001, and Experiment 3 (*M* = 5946, *SE* = 500), *t*(26) = 3.757, *p* < 0.01. No differences were found under complex equations between Experiments 2 and 3, *t*(26) = 0.617, *p* = 0.542. Similarly, under the simple condition (non-carry two addend equations in all three experiments), participants in Experiment 1 performed significantly faster (*M* = 2676, *SE* = 151) than participants in Experiment 2 (*M* = 3154, *SE* = 172), *t*(26) = 2.083, *p* < 0.05, and Experiment 3 (*M* = 3379, *SE* = 287), *t*(19.705) = 2.167, *p* < 0.05, while participants’ performance did not differ between Experiments 2 and 3 as well, *t*(21) = 0.673, *p* = 0.508. In addition, as in the previous experiments, analyzing the accuracy rate in each condition revealed no significant results, all *p*’s > 0.05.

In conclusion, the findings from the joint analysis reveal that participants performed faster after gain stimuli when compared to loss stimuli. This robust effect is present beyond other variables such as difficulty, correctness, or experiment type. Moreover, analysis suggests that when compared to Experiments 2 and 3, Experiment 1 was easier in both the complex and simple conditions.

## General Discussion

The above experiments demonstrate that arithmetic performance can be modulated by emotional gain and loss stimuli. In Experiment 1, an arithmetic equation judgment task was used, with either carry or non-carry equations followed by line drawn faces representing different gaining conditions (gain, neutral, and loss). The findings suggest a robust effect in which participants’ performance in the arithmetic task was faster after gain stimuli when compared to after loss stimuli. Moreover, this effect was found to be larger under the more difficult carry condition. In Experiment 2, the equations’ complexity levels were manipulated by using two vs. three addend equations. Similar to Experiment 1, participants’ performance in the arithmetic task was faster after gain stimuli than after loss stimuli. However, in this experiment the effect did not interact with the complexity level. In Experiment 3, the proportion of correct and incorrect equations was modified. Again, results replicated those of Experiments 1 and 2, suggesting participants’ performance in the arithmetic task was faster after gain stimuli than after loss stimuli. Analyzing all three experiments together again revealed faster arithmetic performance after gain stimuli compared to loss stimuli. These results were found across all experiments and regardless of other factors, such as level of difficulty or equation correctness.

Arithmetic calculations require the involvement of different EF components, such as WM (De Stefano and LeFevre, 2004; Imbo et al., 2007; Raghubar et al., 2010) and the ability to control and inhibit irrelevant information (Passolunghi and Siegel, 2001; Zhang and Wu, 2011). Considering the multiple EF underlying arithmetic performance on the one hand, and the contradicting effect of gain vs. loss stimuli on different EF on the other (e.g., Gilbert and Fiez, 2004; van Steenbergen et al., 2009; Braem et al., 2013; Chiew and Braver, 2014), it is of great importance to not only examine the existence of gain and loss influence on arithmetic performance but also its direction. Our results provide the first evidence for the existence and direction of gain vs. loss modulation on arithmetic performance under different levels of difficulty such as two addends, three addends, carry, and non-carry. All three experiments indicated faster performance after gain stimuli when compared to loss stimuli, suggesting that when performing arithmetic calculation, it is better to use gain stimuli rather than loss stimuli. This can be due to a facilitating effect of the gain stimuli or a hindering effect of the loss stimuli.

Please note that the current gain vs. lost effects are observed although they were randomly presented and were not depended on the participant performance. Due to the fact that gain and loss stimuli are not provided based on one’s behavior, the observed effect can be attribute to the experience involving gain and loss regardless of other potential interfering factors such as control mechanisms assign to adjust behaviors. For example in a recent study, Núñez-Peña et al. (2017), found that participants’ performance in an addition task was slower and more error prone when an error response was made in the previous trial compared to a correct response. In this case, inhibitory self-adjusted mechanisms that come into action in a post error trial might lead to response inhibition hindering both reaction time and accuracy.

While the pattern of results can be attributed to the experience of gain and loss it worth noting that the current gain and loss stimuli was conducted using a combination of “social” gain and loss (i.e., happy or sad face) and monetary gain and loss (gain and lose of money). The use of such combination is well documented in the literature (e.g., Matthys et al., 1998; Kerr and Zelazo, 2004; Yeung et al., 2004; Bunch et al., 2007; Gao et al., 2009; van Steenbergen et al., 2009, 2012). It seems logical to assume that these stimuli have different impacts and each individual is influenced differently by these kind of stimuli as they are affected by different factors such as cultural differences, socio-economic status or gender difference. For example, in a fMRI study by Spreckelmeyer et al. (2009), monetary and social delay tasks were used in order to examine the neural mechanisms underlying reward anticipation in male and female participants. Interestingly, their results show that while male participants reacted faster to monetary reward than social reward, female participants’ reaction to social or monetary reward did not differ significantly. Moreover, while male participants displayed wider network of brain areas sensitive to monetary reward, women displayed the opposite showing wider network of brain areas sensitive to social rewards than man.

Aside from the interesting and robust findings regarding the advantage of gain compared to loss stimuli on arithmetic performance, two more interesting findings require further discussion and will be elaborated next: the interaction between level of difficulty and gaining condition found in Experiment 1, and the effect of the neutral condition compared to the loss or gain stimuli across experiments. As detailed above, a significant interaction was found in Experiment 1 between the effect of gain vs. loss and equation difficulty, as well as a marginally significant interaction between the effect of gain vs. loss and equation correctness. In both cases, the direction of the interaction was such that the effect of gain stimuli compared to loss stimuli seemed to be more pronounced under the more complex and EF demanding conditions, whether these were the equations demanding the carry operation or the incorrect equations condition, requiring extra monitoring. Findings from different studies indicate that the more carry and borrow operations needed in a specific mental calculation, the greater the need for various EF, such as inhibition and WM (e.g., Fürst and Hitch, 2000; Imbo et al., 2007). Similarly, studies examining the processes involved in evaluating correct vs. incorrect arithmetic equations found evidence for increased cognitive demands when involving incorrect equations. For example, in a study by Menon et al. (2002), greater activations were observed in the DLPFC as well as in the left ventrolateral prefrontal cortex during incorrect compared to correct equation solving. It is suggested that this increase in activation represents the additional operations involved in the process of solving incorrect equations, such as maintaining information in the WM while resolving the conflict of the incorrect result (Menon, 2010).

Along with the previously discussed effect of gain and loss on EF, it is not surprising to see more prominent results under more difficult conditions. Nevertheless, note that these interactions were only observed in Experiment 1 and do not seem to reflect a robust pattern, as they do not appear in Experiments 2 and 3 or in the joint analysis of all three experiments. It is possible that the overall difficulty level in Experiments 2 and 3 led to an increased affective modulation of performance even in the simpler condition of these experiments (i.e., two addend equations). Note that the joint analysis reveals that participants indeed performed faster in Experiment 1 compared to Experiments 2 and 3, and that even under the simple condition (i.e., non-carry – two addend equations), performance differed between Experiment 1 and Experiments 2 and 3, such that participants also performed faster in that condition in Experiment 1 vs. Experiments 2 and 3. Hence, it is possible that in Experiments 2 and 3 all conditions were hard enough that the effect of gain and loss stimuli was vivid under all conditions.

Another point to be discussed in the context of the current results is the effect of the neutral condition. Although consistent significant differences were found between positive and negative stimuli across all experiments, the comparisons with the neutral condition seem to differ across experiments. The joint analysis revealed a pattern by which performance after neutral stimuli was between the gain and loss stimuli (i.e., better than loss stimuli though worse than gain stimuli). Nevertheless, this pattern did not reach significance levels. Examining the effect of the neutral condition between the three experiments revealed different patterns. Whereas in Experiment 1 the neutral stimuli seemed to be significantly slower compared to the gain stimuli while not significantly differing from the loss stimuli, in Experiments 2 and 3 the neutral stimuli was faster when compared to the loss stimuli without significantly differing from the gain stimuli. These inconstant results regarding the neutral condition is a study limitation as it does not allow to conclude on the facilitation or the interference components. On the other hand, these inconstant results are consistent with the work of others who performed similar comparisons between neutral and gain or neutral and loss situations, and similarly failed to reach consistent results (see van Steenbergen et al., 2012). It has been argued that when all three conditions appear together, as in this study, the value that the neutral stimulus receives may vary in such a way that it is not perceived as neutral but rather, in each trial, as having non-loss (gain) or non-gain (loss) value (see van Steenbergen et al., 2012). Therefore, it is difficult to produce a consistent and significant pattern of results from a comparison with the neutral condition. As a result, the neutral condition cannot be considered as a reliable reference and therefore comparisons with the neutral condition should be interpreted with cautious.

## Conclusion

The current study bridges two fields of research, one that studies modulation in the form of gain and loss and another studying arithmetic performance. Our results extend the study of gain vs. loss modulation to the important cognitive ability of solving arithmetic problems and specifying the direction of that influence. Arithmetic has an essential role both in our educational years and in daily life. In light of its importance, numerous studies investigated different factors that either improve or hinder arithmetic performance (e.g., Hauser et al., 2013; Park and Brannon, 2014; Praet and Desoete, 2014; Nunez Castellar et al., 2014).

The current study portrays how gain and loss situations differentially affect arithmetic performance. These findings are also of clinical importance and could be taken into account in educational environments. For example, performing a sport contest between pupils in which some lose while others win can also affect the performance of students when solving arithmetic problems in math class. It should be noted that this field of research was scarcely studied so far, but our robust findings might encourage others to further broaden the findings into other arithmetic and mathematical operations as well as to other reward and loss situations.

## Ethics Statement

The study was approved by the ethics committee of the University of Haifa, Israel and was conformed to those standards. Written informed consent was obtained from all participants.

## Author Contributions

Conceived and designed the experiments: LG and RN. Performed the experiments: RN. Analyzed the data: RN. Wrote the paper: LG and RN.

## Conflict of Interest Statement

The authors declare that the research was conducted in the absence of any commercial or financial relationships that could be construed as a potential conflict of interest.
